# The effect of urban-rail station area coverage on city blocks’ epidemic transmission: the case of the rail-supportive city of Beijing, China

**DOI:** 10.3389/fpubh.2025.1588004

**Published:** 2025-05-30

**Authors:** Weitao Zhang, Jingwei Li

**Affiliations:** School of Architecture and Design, Beijing Jiaotong University, Beijing, China

**Keywords:** urban rail, station area coverage, city block, epidemic transmission, mediating effect, moderating effect

## Abstract

**Background:**

From coronavirus disease 2019 (COVID-19) to other human-to-human infectious diseases, the integrative development of rail transport and land use, which is dominated by the theory of the transport–land use feedback cycle, concentrates citizens’ large-scale flow and gathering within the rail station areas (RSAs). This makes RSAs the potential “focal point” of epidemic spread in cities. This study examined the effect of RSA coverage on epidemic transmission in rail-supportive city blocks and further revealed the internal mechanism and potential factors behind the surface effect.

**Methods and results:**

A quantitative empirical analysis was conducted using a typical COVID-19 case in Beijing, China, in 2020, and the statistical analysis method of “a mediating model with a moderating effect” was used, resulting in the following multilayered outcomes: (1) The higher the coverage, the lower the risk, overall, which is different from the general empiricism-based judgment. (2) Behind the total effect, RSA coverage does not directly affect epidemic transmission in blocks, as expected because of the focal point effect on epidemic occurrence possibility. Instead, RSA coverage has a mediating effect on epidemic vulnerability by affecting the residential population size of blocks. (3) There is a strengthening effect on RSA coverage affecting the population size as RSA transport and service levels increase.

**Conclusion:**

These findings have several implications. First, the implementation of contemporary local nonpharmaceutical interventions can be considered to reduce the focal point effect of RSAs and decrease the infectious sensitivity of the block population. Second, the transport–land use integration plays a key role behind the mediating and moderating effects by shaping resident land use and population distribution. Third, the blocks’ primary hospitals, advanced hospitals, municipal roads, and elastic facilities probably provide potential support in reducing blocks’ epidemic risk.

## 1 Introduction

The coronavirus disease 2019 (COVID-19) pandemic, which is one of the world’s largest, has severely tested the ability of cities around the world to respond to major public health emergencies. Global epidemic containment efforts have achieved various results, and we have entered the stage of an in-depth summary and review of experiences. From COVID-19 to other infectious diseases that spread through air and contact, in human-to-human transmission, people are both the virus spreader and the recipient. Therefore, the city has become a hotbed for the spread of infectious diseases because of its essential characteristic of accommodating massive population movement and aggregation (1).

The rail-transport and land-use integrative development mode adopted by many modern cities, which is dominated by the theory of the transport–land use feedback cycle (2), concentrates citizens’ large-scale flow and gathering along the rails, especially within the rail station areas (RSAs), by promoting both high transport accessibility and high development intensity (3). From the viewpoint of epidemic dynamics (4, 5), the rail’s mass traffic flow into RSAs would lead to virus importation (6), and then a chain of local transmission would arise supported by dense population interactivities (7) within RSAs. Importation and local transmission are believed to jointly contribute to the accelerated epidemic occurrence in RSAs and make RSAs a potential “focal point” of epidemic spread. Therefore, city blocks covered by more RSAs may be at a greater occurrence possibility of epidemic. This has become a plausible, empiricism-based judgment put forward by some researchers and policymakers, and it attracts attention in the interdisciplinary field of epidemic transmission and urban studies.

However, according to the United Nations Office for Disaster Risk Reduction (UNISDR), disaster risk is comprehensively assessed based on the occurrence possibility of hazards and the vulnerability of exposed bodies (8, 9). This means that the epidemic risk in city blocks will also be determined by their epidemic vulnerability. For the blocks in a rail-supportive city, RSA coverage may not only affect their epidemic occurrence but also influence their epidemic vulnerability by shaping the blocks’ local demographic–socioeconomic development and built environments. Many studies have validated that rail and RSA development have a significant impact on the population and environment of the surrounding wider areas (10, 11). Many of these dependent variables are important factors in evaluating epidemic vulnerability (12, 13). Meanwhile, studies have found that the extent of impact can be reduced by increasing proximity to transit stations or by decreasing RSA coverage (14).

Moreover, RSAs often have different transport and land-use levels due to diverse integrative development backgrounds and conditions. Under these circumstances, the affecting capacity of RSA coverage, regardless of whether on blocks’ epidemic occurrence or on their vulnerability, may be strengthened or weakened, associated with changes in RSAs’ transport levels and land-use intensities.

**Figure 1:**
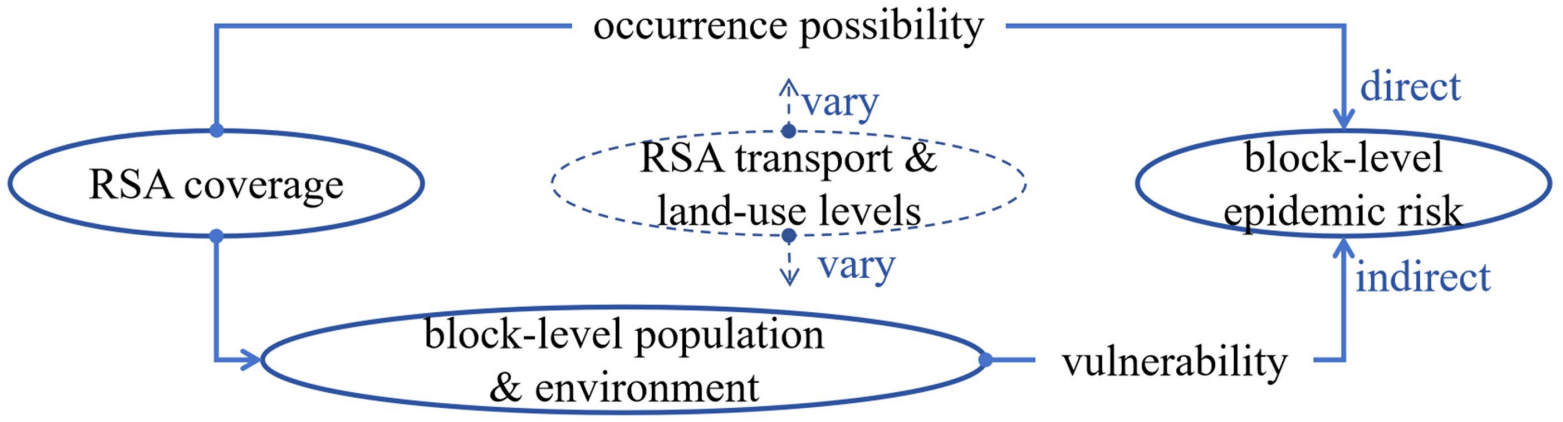
Internal structure of the affecting mechanism.

Above all, according to the theoretical framework of comprehensive disaster risk assessment, the overall impact of RSA coverage on epidemic risk at the city-block level is jointly determined by (1) its direct effect on the occurrence possibility and (2) its indirect effect on the vulnerability through mediating factors such as block-level population and environment. Moreover, the magnitude of these effects may vary depending on RSA characteristics and levels (Figure 1). This affecting mechanism of RSA coverage on blocks’ epidemic transmission has a complex internal structure, which very few existing studies have considered and becomes the core research question of this study. It will make the affecting outcomes much more interesting and probably go beyond empiricism-based judgments. The main purpose of this study is not only to examine whether RSA coverage affects city blocks’ epidemic transmission risk but also to further reveal the internal affecting mechanism behind the surface effect. We considered “city blocks” as the study object and conducted a quantitative empirical analysis by applying the statistical analysis method of “a mediating model with a moderating effect.” We used the COVID-19 case around the Xinfadi Wholesale Market in Beijing, China, in 2020, which is one of the most typical epidemic transmission cases in China. The main steps of this study are as follows: (1) This study tested the total effect of RSA coverage on block epidemic transmission to verify the empiricism-based judgment regarding whether blocks covered by more RSAs show greater epidemic risk. (2) This study examined the direct effect of RSA coverage on epidemic occurrence and its mediating effect on epidemic vulnerability by influencing demographic–socioeconomic development and built environments. (3) This study investigated the moderating effect on the direct and mediating effects separately from the transport and service levels of RSAs.

This study provides insights into RSA coverage affecting city blocks’ epidemic transmission specific to the Xinfadi case, which has reference significance for recognizing its exterior factors, internal mechanism, and spatial features. It contributes to epidemic risk identification and containment resource allocation in city blocks covered by RSAs, and calls attention to the need for balanced planning between development and security in rail-supportive cities in the post-pandemic era.

## 2 Literature review

### 2.1 The integration of transport and land use and the development of urban rail

The integrative development of transport and land use is encouraged in most modern cities based on the theory of the transport–land use feedback cycle to achieve sustainable economic, ecological, and social development goals. From the view of spatial planning, TOD (Transit-Oriented Development) is a classic concept that focuses on the effective integration of transit systems and land use by advocating high-density and high-diversity urban developments around public transportation stations (15–17). Calthorpe (18) identified three TOD characteristics: density with compact growth of dwelling units, population, jobs, and activity sites; diversity with mixed land uses; and design with pedestrian-friendly street networks and high-quality environments. Cervero and Kockleman (19), Curtis, Renne, and Bertolini (20), Ewing and Cervero (21), and Knowles (22) developed Calthorpe’s definition by adding additional characteristics: distance to access transit; destination accessibility; and high-frequency transit services. Up to now, the integration of transport and land use has experienced a significant extension from station area development to urban global development (23, 24). To assess the integration situation and reveal its internal correlation, researchers have applied the node–place (NP) model. This is a classic synergistic analytical model that evaluates node (transport) function and place (land use) function individually and then allows various integration situations to be positioned in the same two-aspect plane for description and comparison (25–27).

The public and mass rail transit system is considered to be the backbone of the city’s transport system (28) and the fundamental travel mode for the future (29). By the end of 2023, 563 cities in 79 countries and territories around the world will have opened urban-rail transit lines with an operating mileage of 43,400 km. For example, 58 cities in mainland China have put into operation 12,169 km of urban-rail transit lines until end of 2024, and more than 32 billion passenger ridership was conducted in 2024, with the mileage accounting for about half of the world’s total and the passenger flow ranking first globally (China Urban-Rail Transit Association 2024). Urban-rail transit is the core transportation type studied in the integration of transport and land use. At present, in addition to planning strategies, there are abundant urban rail-oriented studies covering transport–land use feedback mechanisms (30–32), influencing factors (33–35), and distribution characteristics (36) from station area scale to corridor and network scales (37, 38). China is often viewed as an emerging experimental base for these research and practices because of its rapid urban growth and rail transit development (39). Urban rail transport and land use integration has received powerful policy support and has been shaping much of the physical structure and socioeconomic development in Chinese cities, with an economic purpose–driven model since the 1990s and a shift to a social and ecological purpose–driven model since the 2010s.

### 2.2 RSAs and their influences on surrounding development

RSAs, also known as rail catchment areas or rail service areas, are the earliest main body of TOD studies. They are the spatial anchors of the integrative development of rail transport and land use. For RSA development, researchers define their physical size according to the pedestrian-accessible distance from stations (40, 41). However, case-specific physical sizes vary greatly and are mostly derived using a buffer area from 400 to 2000 m, owing to the lack of a fixed standard for the walking environment and distance and to the different local demographic and geographical characteristics (42). Generally, compared with those in western contexts, RSAs in the Asian context can be smaller because of higher population densities and built-up areas (33). Some studies classify RSAs into several buffers, such as inner (0–500 m) and outer (500–1,000 m) (42), to distinguish the degree of rail catchment capacity and integrative development.

The influence of urban rail development on the demographic–socioeconomic conditions and built environments of areas, which are covered by RSAs to different extents, is the mainstream direction of studies. This means that the influence of the integrative development is always radiated to wider areas through the RSAs, rather than being limited in them. For instance, Yang, Su, and Cao experimentally confirmed that distance to a metro station shows a threshold effect on development intensity, with the effect extending to 1.25 km from the station in his case, and verified that distance to transit stations is the most important predictor of the influence (14), but not the man-defined RSA boundary. Many studies have attempted to evaluate particular changes in local development due to the influence of rail or RSA development. Some studies have shown impacts on local economic outcomes, such as property values, employment, and commercial activities (43–45). Other studies have analyzed other outcomes such as land use (40, 42) and the distribution of public-service amenities (46). However, the same factors sometimes show different results depending on a set of contextual effects, including the station-distance effect (47), RSA transport and land-use features (48), and local pre-existing conditions. Therefore, further studies are required to identify particular differences and discover their spatial heterogeneity. To support this research, the acquisition of multisource data and the application of the statistical analysis and geographic information models are essential. For example, Forouhar found that high- and low-income neighborhoods in Tehran have experienced a heterogeneous model of changes in terms of demographic, housing, and land-use factors (49).

### 2.3 The influence of rails and RSAs on epidemic transmission

Combined with the global context of COVID-19, the cross-study between RSA development and epidemic transmission is becoming a potentially important direction, owing to the consensus that regions with high population density and frequent population mobility and interaction are at high risk of COVID-19. Researchers have explored epidemic transmission within RSAs to verify and elaborate on this consensus. For example, Khare et al. testified that diversity and availability of high-quality transit services effectively spread the virus, whereas population density and public transportation mode of travel were insignificant in his analyzed station areas (50). Jia et al. identified the key stations of the Beijing rail transit network that impact COVID-19 spread according to their route diversity (51). However, more studies have focused on epidemic transmission in wider areas along the rail, which exceed the particular boundary of RSAs, to examine whether proximity to rails or stations is associated with a higher risk of transmission. These wider areas’ epidemic dynamics in terms of virus importation and local transmission have been explored more clearly, which can also indirectly support the focal point effect of RSAs.

Specifically, studies focusing on the accessibility of rail transit impact on COVID-19 spread have demonstrated the virus’s importation. For example, many studies have discussed the role of rail transit in spreading the virus from an epidemic source area by dense traffic flow to a wider area (6). Rail transit, which facilitates thousands of people’s gatherings and interactions within enclosed vehicles and station building every day (52), may further aggravate the risk of virus production and importation to somewhere (53). In addition, studies have explored how locally built environments and demographic–socioeconomic conditions affect the transmission of COVID-19 (54–58). They can be explained for a local transmission chain triggered by importation and indicate local epidemic vulnerability. Many studies have reported that epidemic vulnerability can be assessed by factors such as population size (59) or density (60, 61), built environment attributes (62, 63), and ethnically (64) or socioeconomically vulnerable groups (65–67). It is worth noting that studies have found that neighborhoods with the most infections also had the most commuters (i.e., frontline workers who regularly commute from home to work) (68), which supports the value of rail-oriented epidemic studies because of commuters’ strong dependence on it.

Additionally, the epidemic transmission risk of areas around RSAs or near the rail is not consistent across different studies. This inconsistency is due to various factors such as rail accessibility and complex local conditions, combined with multiple epidemic phases and diverse contemporary local nonpharmaceutical interventions (NPIs) (69–72). Thus, assessing the variety of influence performances is always associated with discussing the role of NPIs.

### 2.4 Previous research gaps and the contributions of this study

Current research on urban rail and RSA development, particularly its impacts on surrounding areas’ populations and environments, is extensive yet predominantly driven by economic, social, and ecological objectives. In contrast, studies examining these impacts through the lens of disaster prevention - especially epidemic containment- remain notably scarce. Within the limited existing research addressing how RSA development influences epidemic transmission in surrounding wilder areas, few have adopted a comprehensive risk assessment framework or systematically deconstructed the multidimensional factors and complex structural mechanisms involved, particularly in relation to the heterogeneous characteristics of RSA development.

This study emphasizes the epidemic containment challenges posed by RSA development at the city-block level. By holistically examining how RSA coverage, features, and levels collectively influence both occurrence possibility and vulnerability, we reveal the underlying complex mechanisms. Our findings will provide empirically grounded, quantitative insights for achieving balanced urban planning that integrates RSA development with epidemic safety.

## 3 Methodology

### 3.1 Study site

Our study focuses on Beijing, the capital of China. Beijing is the first city in China to build an urban-rail transit system (also named as Beijing Subway), and its operating mileage ranks first in China. By the end of 2024, Beijing Subway will have operated 27 lines, with a total mileage of 806 km and a daily passenger volume of 11 million (China Urban-Rail Transit Association 2024). As a typical rail-supportive and rapidly urbanizing city, Beijing has been applying rail-transport and land-use integration mode to solve various urbanization problems and pursue multiple sustainable developments, over the past few decades. The citizens’ massive commute movement and dense aggregation of housing, jobs, and public activities are concentrated within RSAs to different extents, leading to the epidemic transmission risk of city blocks covered by RSAs, which has become a potential concern in academic research and containment decisions during and after COVID-19.

**Figure 2:**
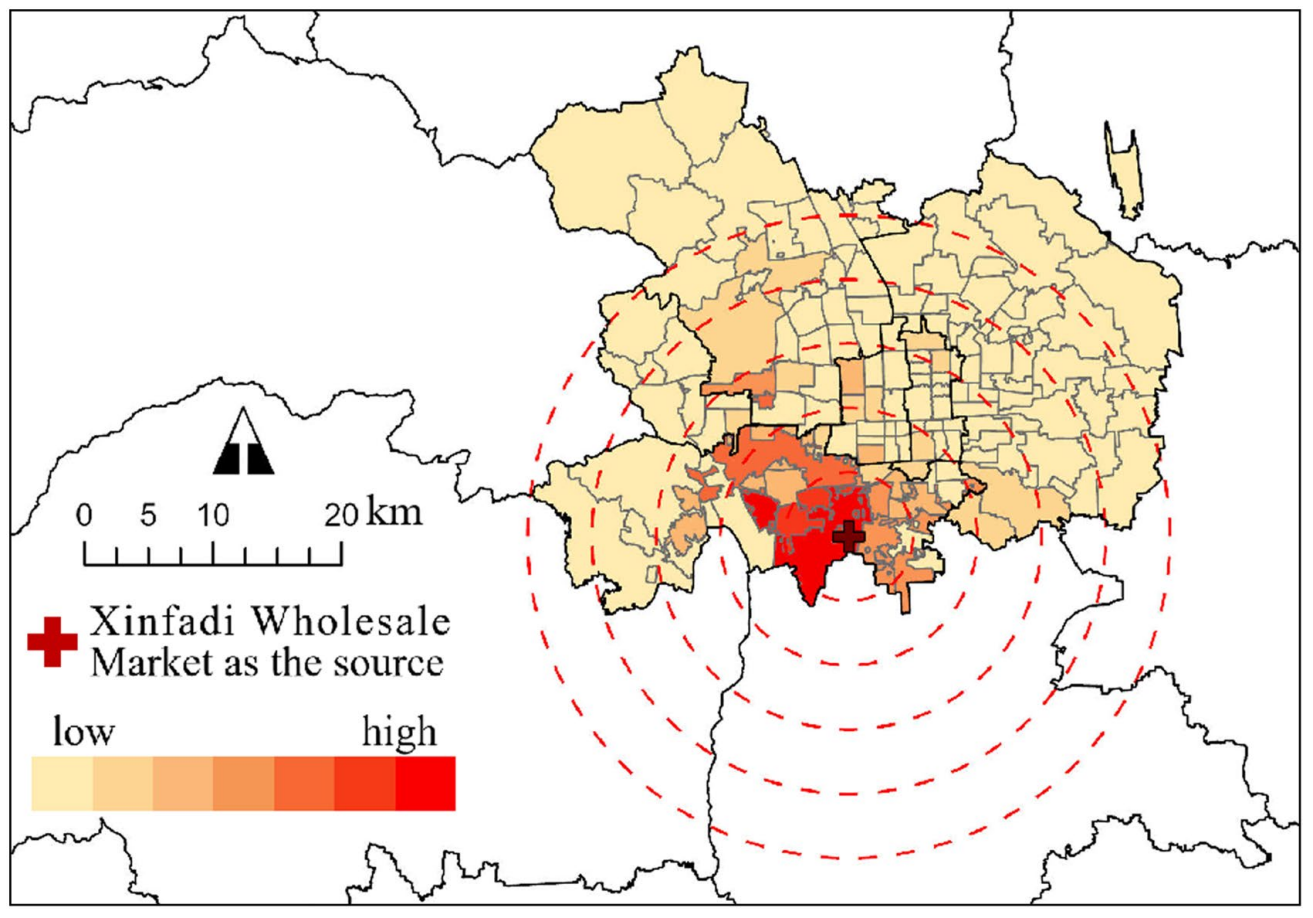
Number of confirmed COVID-19 cases in city blocks.

**Figure 3:**
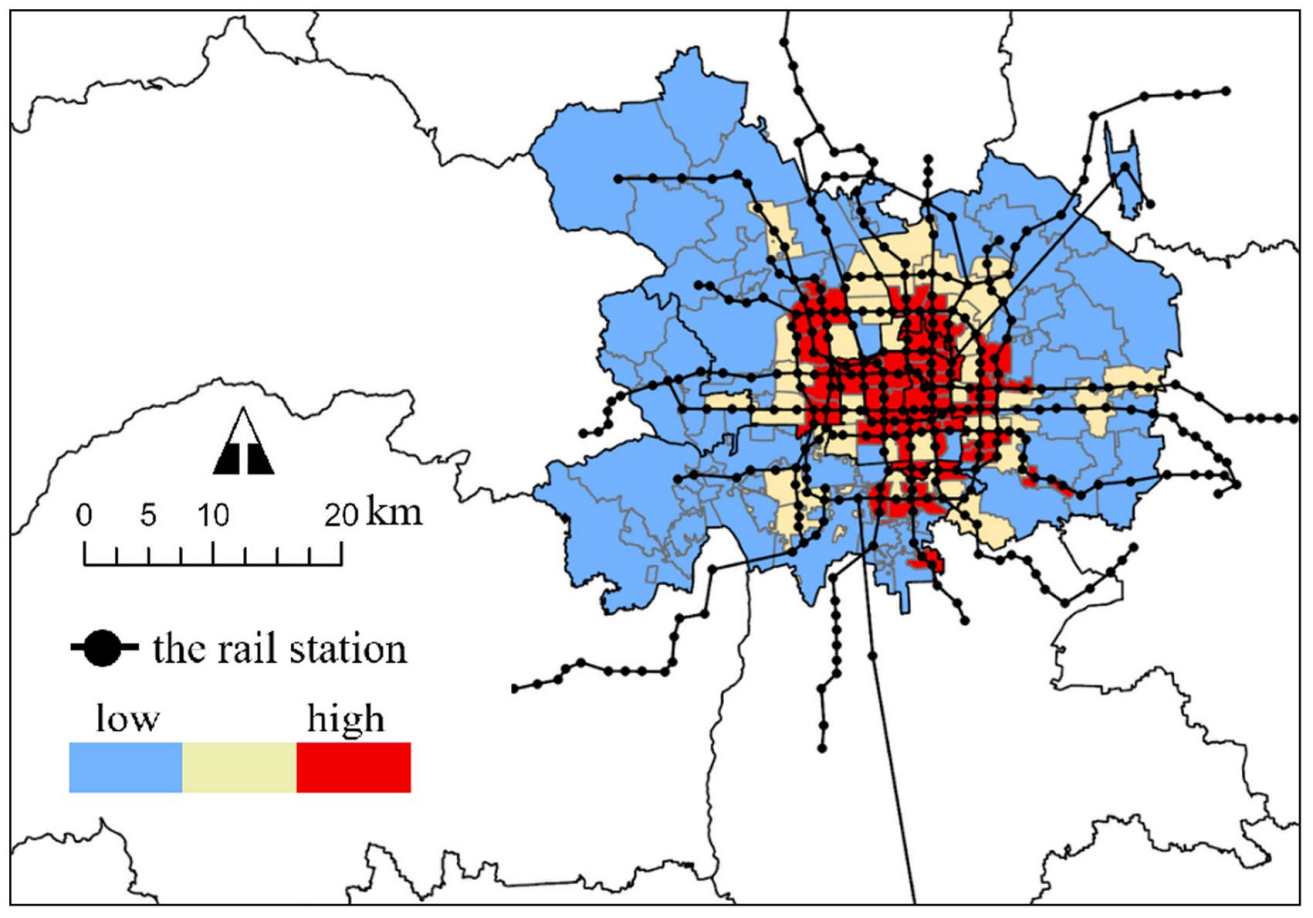
RSA cover rate of city blocks.

This study examines a COVID-19 outbreak in Beijing in 2020, originating from the Xinfadi Wholesale Market, which serves as a key COVID-19 transmission case both globally and in China. As Asia’s largest agricultural wholesale market and cold-chain hub, its high-density crowds and wide mobility range created ideal superspreading conditions, leading to rapid and widespread transmission. This made it a benchmark case cited by Chinese authorities, the World Health Organization, the World Bank, and multiple international think tanks and research institutions. Crucially, its location near three urban rail stations proved significant. Contact tracing revealed that the rail network had become a significant transmission route, thereby providing essential background context for analyzing how RSAs influence epidemic spread at the city block level. After the Xinfadi outbreak, the Beijing municipal government swiftly implemented multiple NPIs and controlled the epidemic for approximately 1 month. In this study, all confirmed COVID-19 cases from June 11 (the day the first confirmed case was identified) to July 6 (the first day that no new confirmed case was identified) were investigated, and all Beijing Subway RSAs and city blocks within Beijing’s main urban area (with a total area of 2337.75 km^2^ and, a population of 10.96 million people) were tested to analyze the effect of Beijing Subway RSA coverage on the epidemic transmission of city blocks, as shown in Figures 2, 3.

### 3.2 Data and measurement of samples and variables

We used various data to measure 13 variables, as shown in Table 1. The selection of variables are based on this study’s theoretical foundation and combined with previous related studies, owing to data availability constraints. The spatial scopes for the measurement of RSAs and city blocks are defined as follows: (1) A 600-meter buffer zone was used to measure RSAs, based on the mobile signaling data analysis by the Beijing Transport Research Center (Annual Report on Passenger Travel Characteristics of Beijing Rail Transit 2021), which shows: (a) a 550-meter median Euclidean walking distance to stations, (b) a 500–600 meter range containing most observations, and (c) a long-tail distribution. The selected threshold balances majority coverage with distributional considerations. (2) Consistent with the boundary of urban administrative management, 133 Jiedaos (the administrative divisions at the same level as townships in China) were selected to represent city blocks, excluding one Jiedao in which the epidemic source was located. Variables were measured in each block sample.

**Table 1:** List of variables.

Role	Variables		Definition	Data source
Dependent	Epidemic transmission risk (Etr)		Number of confirmed cases: 1 = no case; 2 = one case; 3 = more than one case	Data of the Beijing Municipal Health Commission
Independent	RSA cover rate (Rcr)		Cover rate of 600-m buffer of urban-rail stations: 1 = 0–25%; 2 = 25–50%; 3 = 50–100%	Beijing administrative districts map; Beijing Subway data
Mediating (demographic–socioeconomic)	1	Resident population size (Rps)	Resident population number including registered resident population and floating resident population	China’s seventh census data
	2	Registered resident percentage (Rrp)	Percentage of registered resident population in the total resident population	China’s seventh census data
	3	Older adult people percentage (Epp)	Percentage of people over 65 years old in the total resident population	China’s seventh census data
	4	Job–housing deviation index (Jhd)	Ratio of employed population propotion to resident population propotion: 0–1 = residence function dominates; >1 = employment function dominates	Reference (68)
Mediating (built environment)	1	Density of primary hospitals (Phd)	Ratio of primary hospitals to blocks’ area	Beijing POI data
	2	Density of advanced hospitals (Ahd)	Ratio of comprehensive hospitals and infectious disease hospitals to blocks’ area	Beijing POI data
	3	Density of municipal roads (Mrd)	Ratio of municipal roads to blocks’ area	Beijing roads data
	4	Density of elastic facilities (Efd)	Ratio of gardens, squares, community centers, stadiums, and primary and middle schools to blocks’ area	Beijing POI data
Moderating (transport)	1	RSA transport level (Rtl)	Multiply the RSA cover rate by the number of interchange rail lines at the stations	Beijing Subway data
Moderating (land use)	2	RSA service level (Rsl)	Density of restaurants, supermarkets, shopping malls, and leisure services in the RSA coverage area	Beijing POI data
Control	Distance to the epidemic source (Des)		Euclidean distance from each block’s geometric center to the geographical coordinates of the Xinfadi Wholesale Market	Beijing administrative districts map; Beijing POI data

Specifically, the epidemic spread risk in city blocks was assessed using the cumulative number of confirmed COVID-19 cases in each block during the study period. It is assumed that no case means no risk of transmission, one case indicates an accidental risk of transmission, and more than one case represents an obvious risk of transmission. Next, for measuring RSA coverage in each block, there may be more than one RSA covering a single block.

For the demographic–socioeconomic conditions of city blocks, the resident population number and the percentage of older adult people (over 65 years old) and marginalized people in each block were counted. These figures reflect the population size (59), individual immunity (73), and individual prevention ability against viruses (74–76), all of which are important factors for vulnerability assessment applied in previous research. The nonregistered resident population, compared with the registered resident population, has limited socio-economic security and a weak community-governance connection and can be seen as a type of marginalized population. We also applied the job–housing deviation index (JHDI) to assess the gross dominant function of each block, which is measured by the ratio of the employed population proportion to the residential population proportion (77). When the value equals 1, employment and residential functions are balanced. A value of more than 1 indicates that employment is the dominant function in this block. Otherwise, the residence function dominates. A high JHDI can reflect the greater cross-transmission risk in a mixed job–housing environment.

For the built environment of blocks, the medical and sanitary conditions, which are decisive supporting factors for epidemic containment in present studies and practices, were assessed in two levels. At the lower level, the density of primary hospitals in each block was counted to reflect the capacity of primary triage for epidemic-related medical treatment. At the higher level, the density of comprehensive and infectious disease hospitals in each block shows the high standard, large-scale centralized treatment capacity. The municipal road density of each block was used to assess the automotive accessibility of relief activities and supplies. It also indicates the capability of physical separation in population and construction concentrated areas, which will support spatial grid-precise containment and quarantine (78). Moreover, we measured the density of “elastic facilities” in each block, including community centers (79), primary and middle schools (80), stadiums (81, 82), and gardens and squares (83). Many studies have proposed that these spaces can be rapidly and cost-effectively converted into multi-use emergency sites following an epidemic outbreak. As such, they have great and important potential to fill the gap in medical and non-medical interventions when there is a rapid surge of patients over a short period of time. These spaces can be used to temporarily accommodate nucleic acid testing, quarantine observation, medical assistance, vaccination, storage and distribution of materials, and various livelihood support when professional facilities are inadequate (84, 85). In this paper, we measured their overall auxiliary capacity for epidemic containment. Above all, the high value of these environmental variables is expected to enhance the containment capacity and reduce vulnerability to epidemic spread.

Furthermore, we multiplied the RSA cover rate of each block by the number of transfer lines at the stations to represent the RSA transport level. Meanwhile, the density of indoor and large public-service facilities (including restaurants, supermarkets, shopping malls, and leisure services) in the RSAs in each block was counted to show RSAs’ service level, in terms of land-use intensity in one particular aspect. On one hand, RSAs’ high transport and service levels may result in a strong performance of RSAs’ focal point effect, with a higher virus importation and a heavier virus local transmission within RSAs. On the other hand, these two variables with high values may be associated with a higher shaping capacity on the population and environmental development of covered blocks.

Finally, because the Xinfadi case is a typical point-outbreak epidemic, the Euclidean distance from each block’s geometric center to the initial epidemic source (the geographical coordinates of the Xinfadi Wholesale Market) was used as a control variable. The Euclidean distance is commonly used as a proxy measure of personal exposure to emission sources (86), based on Tobler’s first law of geography, which states that near things are more related to each other.

### 3.3 Analytical strategy

**Figure 4:**
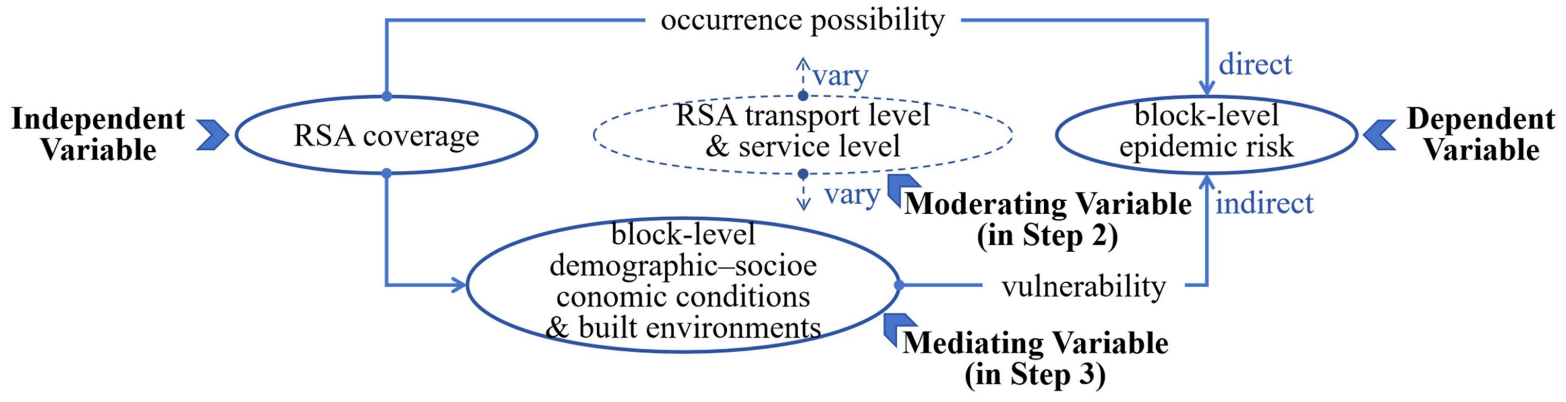
Variable design of the affecting mechanism.

We applied “a mediating model with a moderating effect” to explore the extent to which the effect of independent variable X on dependent variable Y is clarified by the mediating variable (87) and whether this effect’s degree is further affected by the moderating variable (88), as shown in Figure 4. More specifically, the independent variable (RSA coverage) is a three-category variable (1 = 0–25%, 2 = 25–50%, and 3 = 50–100%); the dependent variable (epidemic transmission risk) is a three-category variable (1 = no confirmed case, 2 = one confirmed cases, and 3 = more than one confirmed cases); and the eight mediating variables (demographic–socioeconomic conditions and built environment), two moderating variables (RSAs’ transport level and service level), and one control variable (distance to the Xinfadi epidemic source) are all continuous variables. This model was implemented using the causal steps approach in SPSS 27: (1) test the effect of RSA coverage on blocks’ epidemic risk by ordered logistic regression; (2) examine the effect of both RSA coverage and blocks’ local conditions on blocks’ epidemic risk by ordered logistic regression, after proving that the influence of RSA coverage on the blocks’ demographic–socioeconomic conditions and building environments by linear regression; and (3) separately analyze the interactive effect of RSA coverage with RSA transport and service levels on the blocks’ particular conditions or epidemic risk by linear regression.

To prove that the data applied in the model are effective, for ordered logistic regression, the pretests of model fitting information and parallel lines were passed. In addition, pretests for linear regression, including R square, Durbin–Watson, ANOVA, and VIF, were satisfied. The tabular outcomes of these tests are not shown because of the limited space of the paper.

## 4 Results

### 4.1 Spatial distribution characteristics of variables

**Figure 5:**
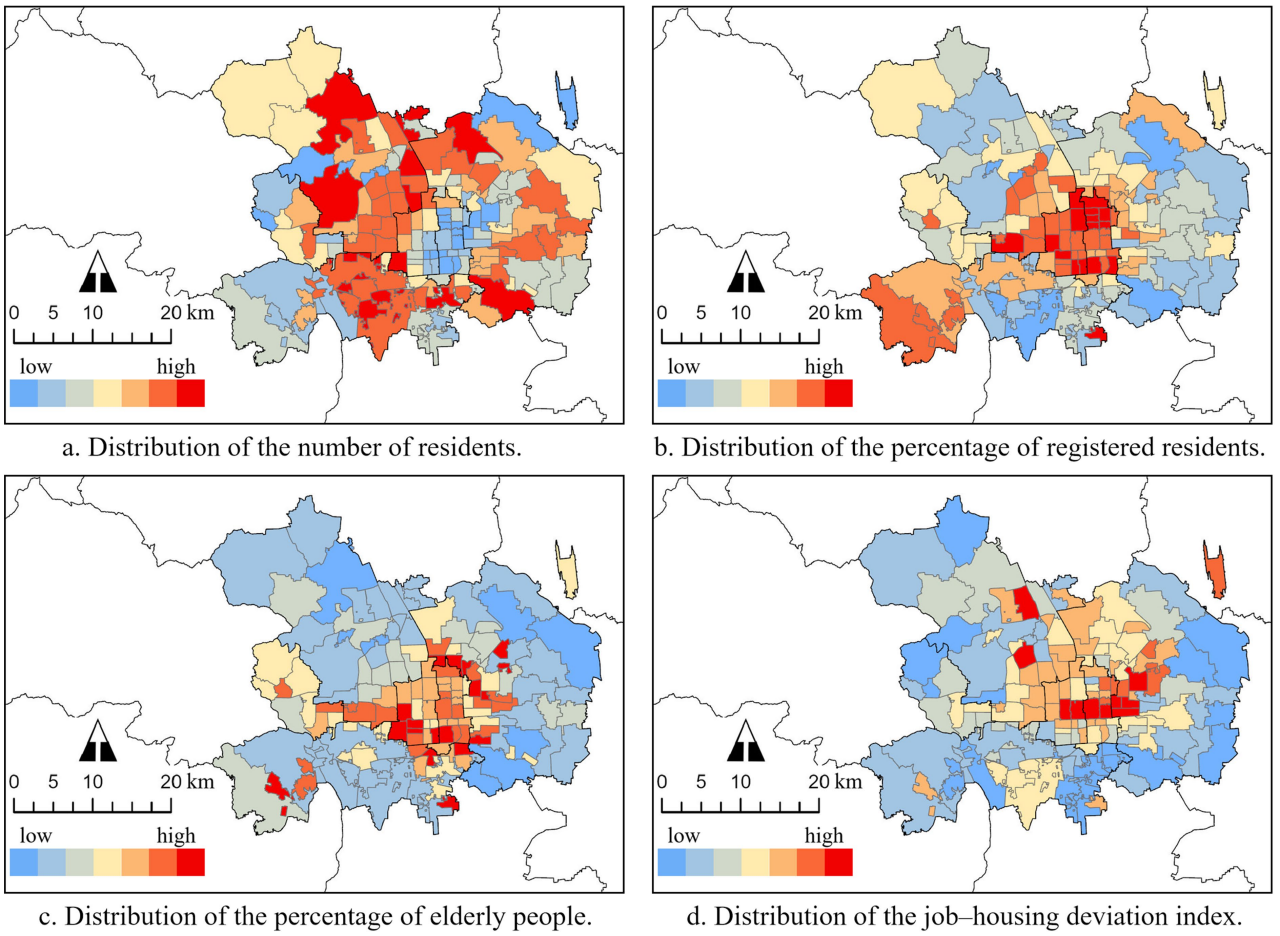
Distribution of demographic and socioeconomic conditions of city blocks.

The spatial distributions of eight variables representing the blocks’ local conditions are shown, using the classification of natural break points method in ArcGIS pro. Several detailed characteristics can be extracted from these maps. The blocks’ resident population (Figure 5a) exhibited an obvious ring shape with a high number in the inner suburb but a low number in the central area and the outer suburb. This is because the dominant function of Beijing’s city center is administrative office, financial service, cultural tourism, and heritage protection. In addition, the city construction of the outer suburb is not yet mature. By contrast, the large proportion of residential land and mature real estate development is much more distributed in the inner suburb.

Both the percentage of the registered residents (Figure 5b) and older adult people (Figure 5c) showed a decrease from the central area but with several high patches in the outer suburb. It is reasonable that the distribution of the long-term resident population is associated with the urban construction’s gradual expansion from the city center. At the same time, there are long-term resident populations brought by long-established enterprises located in the outer suburb owing to low land prices and sufficient land availability. In addition, compared with registered residents, a higher concentration of older adult people is found between the central area and the inner suburb. This may be because urban services are more perfect in these areas than in the suburbs, and living spaces are more spacious than in the city center, which can attract more retired people to choose a residence. The JHDI (Figure 5d) also exhibited a decrease from the central area, in addition to two focal directions, including the IT industrial district in the northwest and the CBD district in the middle east.

**Figure 6:**
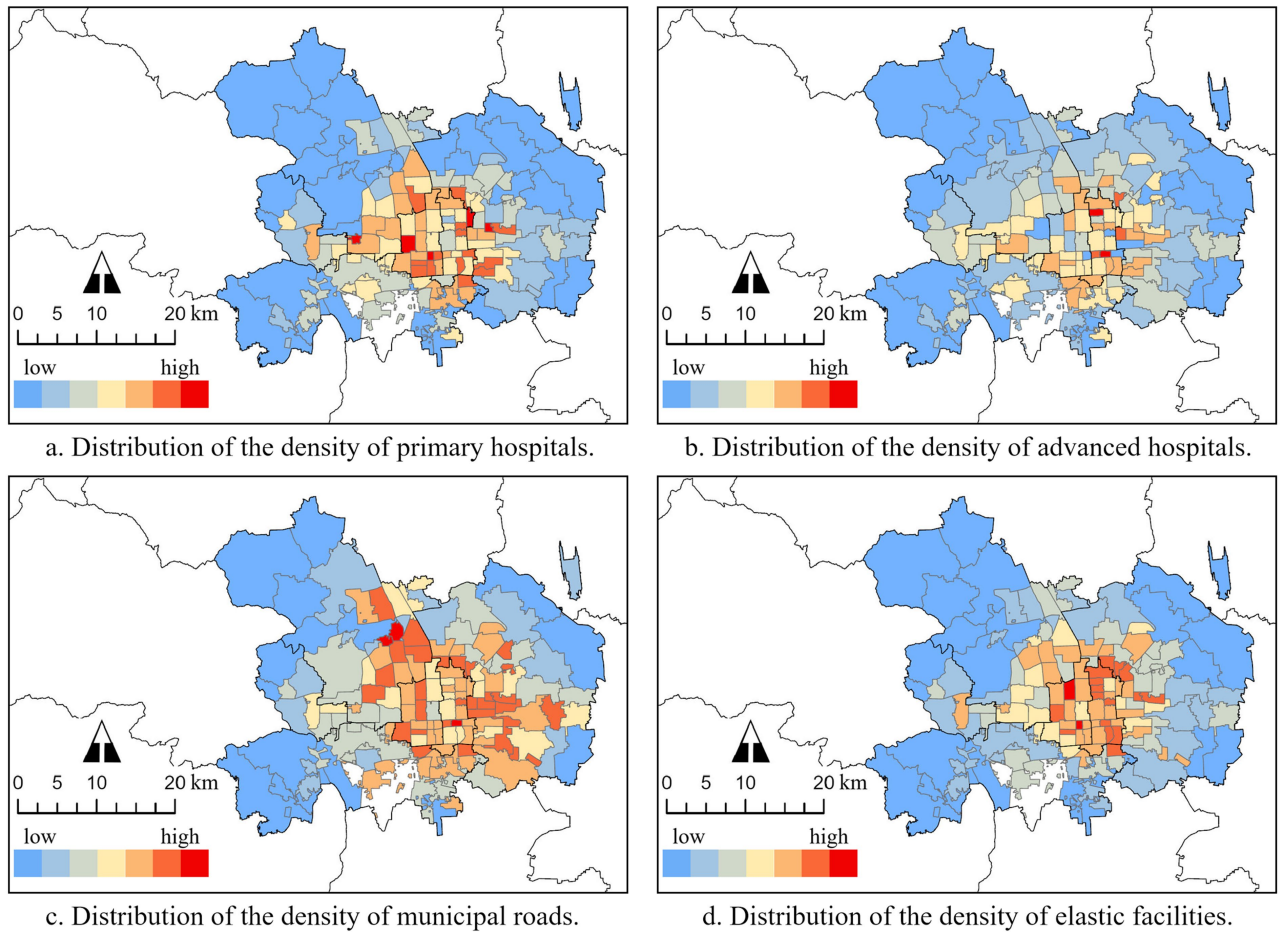
Distribution of built environments of city blocks.

All the densities of blocks’ primary hospitals (Figure 6a), advanced hospitals (Figure 6b), municipal roads (Figure 6c), and elastic facilities (Figure 6d) decrease from the central area but with various degrees of heterogeneity. This can be explained by the central place theory (89). Meanwhile, we can observe that the imbalance of the distribution of public resources exists in both local and wide areas.

Comparing the distribution of the RSA cover rate, which also exhibited a decrease from the center, associated with Beijing Subway’s network of grid-shaped inward and ray-shaped outward, it appears that spatial correlations exist between each variable and RSA coverage. However, further statistical analysis is needed.

### 4.2 Total effect of RSA coverage on blocks’ epidemic risk

The result from Step 1 analysis shows that the total effect of RSA coverage on the epidemic transmission risk of blocks is significant. However, the outcome of higher coverage resulting in lower risk is much different from the empiricism-based judgment and related supporting studies. Specifically, Table 2 shows that the probability of the risk rising one level for high-coverage blocks is 0.182 times that of low-coverage blocks. In addition, the mid-coverage is 0.335 times that of low-coverage blocks, but this is not significant. This unexpected outcome implies that some indicators other than the focal point effect of RSAs play more significant role in affecting blocks’ epidemic transmission. This will be further explained by the next-step mediating effect examination. Furthermore, the distance from the blocks to the Xinafadi epidemic source negatively impacts the blocks’ risk level, consistent with the general judgment and demonstrating the necessity of controlling for this variable. When the distance increases 1 km, the probability of the risk rising one level is 0.716 times that of its previous value in a significant way.

**Table 2:** Parameter estimate.

Independent variable		*β*	OR	*p*	OR 95% CI
Threshold	[Etr = 1]	−3.441	0.032	0.000	0.006 ~ 0.158
	[Etr = 2]	−2.154	0.116	0.004	0.026 ~ 0.511
Location	Des	**−0.334**	**0.716**	**0.000**	0.636 ~ 0.806
	[Rcr = 3]	**−1.704**	**0.182**	**0.005**	0.055 ~ 0.604
	[Rcr = 2]	−1.094	0.335	0.096	0.092 ~ 1.213
	[Rcr = 1]	0^a^	-	-	-

Correlation function: Logit.

a This parameter is the reference category, therefore it is set to zero. The variables with statistical significance are highlighted in bold.

By comparing the prediction results of the regression model with the actual data (Table 3), it is found that the total accuracy rate is 84.21%, indicating that the variable construction in this model has a good performance. Specifically, the prediction for no risk is very accurate, whereas that for the obvious risk is acceptable. In addition, the prediction for the accidental risk shows underperformance. In practice, recognizing no- and obvious-risk units is actually more important.

**Table 3:** Etr predicted response category crosstabs.

Model			Predicted response category		Total
			1	3	
Etr	1	Counting	103	2	105
		Ratio	**98.1%**	1.9%	100.0%
	2	Counting	12	2	14
		Ratio	85.7%	14.3%	100.0%
	3	Counting	5	9	14
		Ratio	35.7%	**64.3%**	100.0%
Total		Counting	120	13	133
		Ratio	90.2%	9.8%	100.0%

The variables with statistical significance are highlighted in bold.

### 4.3 Mediating effect behind the total effect

The Step 2 analysis first indicated that the shaping influences of RSA coverage on all eight factors of the demographic–socioeconomic conditions and built environments of the blocks are significant (Table 4). Therefore, all of them will be applied as candidate mediating factors in the mediation examination. Seven factors (except the residential population) are positively influenced by RSA coverage. We can see that the influence coefficients of higher-coverage blocks are often higher than those of lower-coverage blocks, demonstrating that the influence capacity decreases as the coverage decreases. In contrast, for the residential population, the population of high-coverage blocks is 2.028 times less than that of low-coverage blocks, while the population of mid-coverage blocks is 2.396 times more than that of low-coverage blocks. There is an interesting fluctuation in the residential population across high-, mid-, and low-coverage blocks.

**Table 4:** Coefficients.

Models	Rps		Rrp		Epp		Jhd		Phd		Ahd		Mrd		Efd	
	B	*p*	B	*p*	B	*p*	B	*p*	B	*p*	B	*p*	B	*p*	B	*p*
Constant	9.051	0.000	0.407	0.000	0.161	0.000	0.678	0.020	1.365	0.000	0.862	0.005	10.730	0.000	2.192	0.000
Des	−0.052	0.412	−0.002	0.266	**−0.002**	**0.001**	**0.031**	**0.044**	**−0.039**	**0.002**	−0.027	0.098	−0.050	0.327	−0.038	0.194
[Rcr = 3]	**−2.028**	**0.029**	**0.140**	**0.000**	**0.039**	**0.000**	**1.268**	**0.000**	**1.138**	**0.000**	**1.023**	**0.000**	**5.812**	**0.000**	**4.003**	**0.000**
[Rcr = 2]	**2.396**	**0.018**	**0.066**	**0.008**	**0.020**	**0.011**	**0.570**	**0.020**	**0.625**	**0.001**	**0.579**	**0.024**	**4.442**	**0.000**	**2.047**	**0.000**

The variables with statistical significance are highlighted in bold.

Subsequently, we put both RSA coverage and blocks’ local conditions into the regression model to compare them with Step 1 outcomes, as shown in Table 5. The results indicate that, except the control variable still significantly negatively affecting the epidemic risk of block, only the residential population size of blocks exhibits a significantly positive effect on the risk. When the population increases 10,000, the probability of the risk rising one level is 1.215 times that of its previous value. In contrast, the RSA coverage, as well as the other indicators, does not show a significant effect on risk anymore.

**Table 5:** Parameter estimate.

Model		*β*	OR	*p*	OR 95% CI
Threshold	[Etr = 1]	−3.207	0.040	0.152	0.001 ~ 3.248
	[Etr = 2]	−1.762	0.172	0.426	0.002 ~ 13.118
Location	Des	**−0.371**	**0.690**	**0.000**	0.596 ~ 0.799
	Rps	**0.195**	**1.215**	**0.008**	1.051 ~ 1.404
	Rrp	2.114	8.281	0.577	0.005 ~ 14016.633
	Epp	−8.127	0.000	0.491	0 ~ 3335055.904
	Jhd	0.222	1.249	0.532	0.623 ~ 2.504
	Phd	0.302	1.353	0.502	0.56 ~ 3.261
	Ahd	0.268	1.307	0.359	0.737 ~ 2.316
	Mrd	−0.128	0.880	0.287	0.695 ~ 1.114
	Efd	−0.065	0.937	0.746	0.631 ~ 1.391
	[Rcr = 3]	−1.327	0.265	0.135	0.046 ~ 1.514
	[Rcr = 2]	−1.194	0.303	0.142	0.062 ~ 1.489
	[Rcr = 1]	0^a^	—	—	—

^a^This parameter is redundant, therefore it is set to zero.The variables with statistical significance are highlighted in bold.

From Table 6, it can be concluded that behind the significant total effect of RSA coverage on blocks’ epidemic risk is a “fully” mediating effect. Specifically, RSA coverage cannot directly affect blocks’ risk but can play a mediating role through influencing the residential population size of blocks. This is a further confirmation of Step 1 that the focal point effect of RSA does not work as expected. More interestingly, although the residential population is the only significantly and positively mediating factor that plays a full role, the change in the total effect of RSA coverage on blocks’ risk is not completely consistent with the change in residential population. This subtle detail is worth further discussion.

**Table 6:** Summary and comparison.

Model	Etr		Rps		Etr	
	*β*	*p*	*β*	*p*	*β*	*p*
[Rcr = 3]	**−1.704**	**0.005**	**−2.028**	**0.029**	−1.327	0.135
[Rcr = 2]	−1.094	0.096	**2.396**	**0.018**	−1.194	0.142
[Rcr = 1]	0^a^	—	0^a^	—	0^a^	—
Rps					**0.195**	**0.008**

a This parameter is redundant, therefore it is set to zero. The variables with statistical significance are highlighted in bold.

### 4.4 Moderating effect on the mediating effect

In Step 3, we tested only the moderating effect of RSA transport and service levels on the indirect influence of RSA coverage on the residential population size of blocks, as shown in Tables 7, 8, rather than on the direct influence of RSA coverage on the blocks’ epidemic risk. This is because the residential population has a fully mediating effect on the total effect (as verified in Step 2). Because the interaction term of RSA coverage and RSA transport level is statistically significant, there is a significant strengthening effect on RSA coverage affecting the population as the transport level increases. Based on Step 2 results, this implies that compared with the population of low-coverage blocks, the population of blocks with high coverage of transport-hub RSAs will be much lower. In addition, the population of blocks with a mid-coverage of transport-hub RSA will be much higher. Meanwhile, the interaction term of RSA coverage and RSA service level is significant only when comparing high- and low-coverage blocks. This illustrates that service-center RSAs will further decrease the population of high-coverage blocks, and indicates that the strengthening effect of RSA service is space-limited compared with that of RSA transport.

**Table 7:** VIF test.

Model		Unstandardized coefficients		Standardized coefficients	*t*	Sig.	Collinearity statistics	
		B	Std. Error	Beta			Tolerance	VIF
1	(Constant)	10.318	1.329		7.762	0.000		
	Rtl	−3.614	1.329	−0.307	−2.720	0.007	0.572	**1.749**
	Rsl	0.008	0.008	0.117	1.059	0.291	0.596	**1.678**

Dependent variable: Rps. The variables with statistical significance are highlighted in bold.

**Table 8:** Coefficients.

**Models**	**B**	***p***	**Models**	**B**	***p***
Constant	6.483	0.000	Constant	7.747	0.000
Des	−0.020	0.759	Des	−0.026	0.702
[Rcr = 3]	−6.034	0.020	[Rcr = 3]	−2.156	0.044
[Rcr = 2]	−3.493	0.152	[Rcr = 2]	1.845	0.100
Rtl	15.856	0.011	Rsl	0.017	0.177
[Rcr = 3]*Rtl	**−19.888**	**0.003**	[Rcr = 3]*Rsl	**−0.029**	**0.067**
[Rcr = 2]*Rtl	**−21.422**	**0.010**	[Rcr = 2]*Rsl	−0.019	0.318

If the coefficient’s directions of the principal and interaction variables are consistent, the moderating variable has an increasing effect on the mediating effect. Conversely, the effect of moderating variable is decreasing. Dependent variable: Rps. The variables with statistical significance are highlighted in bold.

## 5 Discussion

### 5.1 The inverse effect of RSA coverage due to NPIs and blocks’ residential population

According to the results of the quantitative empirical analysis of the Beijing Xinfadi case, first, RSA coverage does not significantly affect epidemic transmission in blocks directly and positively. This finding differs from the empiricism-based judgment that higher RSA coverage may result in higher risk due to RSAs’ focal point effect. Specifically, in our case, a reasonable inference can be made that the multiple contemporary local NPIs mitigated RSAs’ focal point effect to a valid extent. After Xinfadi’s epidemic outbreak, the Beijing municipal government quickly closed the Xinfadi Market and contained the source outbreak through a chain, from epidemiological investigation to nucleic acid tests, isolation, and treatment (78). Meanwhile, they implemented residential community closed management, suspended gathering activities, and controlled the rail load rate based on to the classification principle (i.e., to identify and classify existing risks of blocks by assessing their number of confirmed cases and then to adjust the strictness of NPIs according to the risk level) (90). These NPIs limit the rail’s mass traffic flow into RSAs and the dense population interactivities within RSAs, accordingly restricting the epidemic occurrence in RSAs and thus threatening blocks covered by RSAs.

Second, the high positive correlation between the blocks’ epidemic transmission and residential population size is consistent with numerous existing studies. However, the correlation with the older adult and marginalized populations, who are more susceptible to infection due to individual physiological conditions and socioeconomic deprivation, respectively, as suggested in many studies, is not shown in our study. This may result from the home-entry services of information investigation, infection control publicity, subsistence material support, and psychological counseling, applying big data and community governance during the Xinfadi case. Moreover, the degree of productive activities mixing in residences also did not show significant effect in our study. This indicates that measures such as working from home, social distancing, and community closed management applied by Beijing played an important role.

Overall, the Beijing Xinfadi case is recognized as the most typical example of China’s NPI response to COVID-19 (90). In this study, NPIs probably not only mitigated RSAs’ epidemic occurrence risk to blocks but also reduced the epidemic vulnerability risk in terms of sensitive groups within blocks. As a result, only the blocks’ residential population became the decisive factor positively affecting the blocks’ epidemic there.

### 5.2 The key role of transport–land use integration behind blocks’ residential population distribution

The residential population size is the only decisive factor affecting the blocks’ epidemic transmission, but its spatial distribution hinges on the key role of transport–land use integration in shaping built environments and population activities, both inside and outside RSAs. In many studies and practices, RSAs are divided into multiple buffers to distinguish inner, outer, and outside development zones. Generally, the RSA inner buffer is often characterized by higher building density, a higher plot ratio, and a higher proportion of commercial and public service land use, due to high accessibility and high land prices. By contrast, the plot ratio in the RSA outer buffer tends to be lower, but the proportion of land use for residents increases dramatically, as it gets rid of the space crowding of large-public facilities. Areas outside RSAs show a much lower plot ratio, but the various types of land use is no longer limited owing to rich land resources. Referring to these features, the outcomes in which the resident population is lowest in high-coverage blocks and highest in mid-coverage blocks can be explained to a large extent.

When considering the level and function of RSAs, the strengthening effect of developer RSAs on resident population distribution can also be explained. For transport-hub RSAs, because of the large land requirement to support the large scale of transportation facilities and comprehensive transportation connections, the proportion of residential land use in the RSA inner buffer is further decreased. However, the outer buffer will attract much more residential real estate development due to advanced commuter accessibility. For service-center RSAs, in the inner buffer, the diverse service facilities are often adjoined with open spaces and landscape facilities, leading to rarer land resources and higher land price. Therefore, this lowers the proportion of residential land use associated with resident population even further. However, compared with the outer buffer of transport-hub RSAs, the outer buffer of service-center RSAs shows less attraction for resident population because the daily need for leisure accessibility is much lower than that for commuting accessibility in Beijing. This may explain why the strengthening effect of RSA service is space-limited compared with that of RSA transport.

In summary, although RSA coverage does not directly affect the epidemic transmission risk of city blocks, its transport–land use feedback attribute and functional differences indirectly work by guiding resident land use and population distribution.

### 5.3 The potential function of blocks’ built environments on blocks’ epidemic risk

Blocks’ built environments including the densities of primary hospitals, advanced hospitals, municipal roads, and elastic facilities reflect the local emergency capacity to an epidemic. Although they were not proven to demonstrate a significant mediating effect on RSA coverage affecting blocks’ epidemic risk, their subtly potential mediating effect can be revealed by analyzing in-depth the outcomes across multiple-step regressions. Specifically, in Step 2, we can see that the residential population of mid-coverage blocks is significantly higher than that of low- and high-coverage blocks in sequence. Meanwhile, the resident population has a fully decisive positive effect on the blocks’ epidemic risk. Therefore, for a reasonable inference, the risk for mid-coverage blocks would be the highest, and the risk for high-coverage blocks would be the lowest. However, in Step 1, the epidemic risk of mid-coverage blocks is not the highest and is also not significant. Based on this, we can modify the inference as the following: the decisive effect of resident population is reduced in the total effect of mid-coverage on blocks’ epidemic risk. This is probably because of the more perfect primary hospitals, advanced hospitals, municipal roads, and elastic facilities in mid-coverage blocks compared with low-coverage blocks. These important facilities for epidemic containment may provide support to reduce the epidemic risk of mid-coverage blocks. Which specific facility plays a more significant role still needs further research.

Overall, although the results indicate that the densities of city blocks’ primary hospitals, advanced hospitals, municipal roads, and elastic facilities are not significant mediating factors for RSA coverage affecting blocks’ epidemic transmission, some of them are inferred to have a potential mediating effect. Moreover, the distribution of these facilities is still closely related to the role of transport–land use integration around RSAs in shaping urban built environments.

## 6 Conclusion

This study conducted an empirical analysis, using a typical COVID-19 case in Beijing, China, to examine the effect of RSA coverage on the epidemic transmission risk of city blocks and reveal its internal mechanism and potential factors. We incorporated 13 variables into “a mediating model with a moderating effect,” resulting in multilayered outcomes.

A prominent insight from this study is that the higher the coverage, the lower the risk, overall. Findings from the mediating effect analysis indicate that RSA coverage does not directly affect blocks’ epidemic risk, as expected because of the focal point effect. Instead, it has a fully mediating effect by affecting blocks’ residential population size, which is the only significantly affecting factor behind the total effect. Moreover, there is a strengthening effect on the influence of RSA coverage on population size as RSA transport and service levels increase. First, implementing rapid, rigorous, and precise NPIs effectively reduced the focal point effect of RSAs and decreased the infectious vulnerability of the block population. Second, although RSA coverage does not directly affect city blocks’ epidemic risk, its transport–land use feedback attribute plays a key role in mediating and moderating the effects of the resident population on blocks’ risk. Its shaping capacity on urban built environments, specifically to resident land use, deeply influences the distribution of resident population within and outside RSAs (across ordinary, transport-hub, and service-center RSAs). Therefore, the residence-based epidemic risk in a block can be pre-estimated to a certain extent through the development characteristics of RSA, including coverage, transport level, and service level. Third, by comparing total effect analysis and mediating effect analysis, the subtle differences indicate that even the blocks’ primary hospitals, advanced hospitals, municipal roads, and elastic facilities do not show a significant effect, some of them probably provide potential support in reducing blocks’ epidemic risk. As a result, although the theory of transport–land use feedback and TOD principles do not specifically emphasize enhancing public services and road infrastructure from the perspective of epidemic containment, greater attention should be paid to the rational expansion of scale, quantity, and standards of these facilities when they are integrated with residential development. It will support a balance between epidemic safety and RSA development.

However, it should be acknowledged that the Beijing and Xinfadi cases are unique and that potential differences may arise compared to other regions and epidemic cases. First, Beijing is characterized by rapid and large-scale urban rail development. Compared to conventional busses, rail transit offers much higher accessibility, which establishes a stronger feedback cycle with land use around its stations. This supports the “focal point” of epidemic spread within RSAs and allows RSA development to have a significant impact on the population and environment of surrounding areas. In contrast, in cities with only conventional bus systems or low levels of rail development and usage, such impacts are likely to be significantly smaller. Second, the Xinfadi case is a landmark in China’s epidemic containment efforts, characterized by rapid response, precise epidemiological investigation, and large-scale screening. However, this containment approach may not be applicable to other countries and regions. Under slower and more moderate Non-Pharmaceutical Interventions (NPIs), the “focal point” effect of RSAs may be more pronounced, and the multiple sensitivities of city-block populations may play a more significant role. Together, these factors would lead to a more complex set of influences on block-level risks.

These findings have several implications. First, for the generally empiricism-based judgment that blocks covered by more RSAs or closer to rails may be in the greater risk of epidemic, it would be different from case-specific studies because of various NPIs implemented and local particular conditions. Meanwhile, our study proves that the role of spatial planning oriented by transport–land use integration in affecting and containing epidemic risk exists and should be seriously acknowledged, even though probably in an significantly indirect way (such as through influencing population distribution) or a statistically insignificant way (such as through guiding the distribution of emergency-supportive facilities). The configuration of primary hospitals, advanced hospitals, municipal roads, and elastic facilities should be refined in accordance with the characteristics of block-level residential population distribution influenced by RSA development. Particular attention should be paid to enhancing the facility construction in RSA mid-coverage blocks, especially those in transport hub-type RSAs. On this basis, the scale, quantity, standards, and personalized services of facilities should be adjusted according to the epidemic sensitivity features influenced by block-level population structure. In conclusion, the combination of NPIs and spatial planning can contribute to prejudging and containing the epidemic risk. To conduct a cross-study analysis between them will be a great potential direction for future research.

This study has several limitations. First, it does not distinguish between epidemic phases and NPI timing because the NPIs in this study case started much quickly and the epidemic was short. Second, based on the median Euclidean walking distance to Beijing Subway stations from the empirical survey, a 600-meter buffer zone was used to measure all RSAs. This approach may not accurately represent the actual catchment areas incorporating different RSA types. However, we used transport and service levels as additional variables to further examine the moderating effects of different RSA characteristics on block-level risk. Lastly, in this study, the number of confirmed cases (representing epidemic risk) was counted according to their place of residence, rather than their place of infection or contact. This approach helps to reveal that RSA coverage significantly influences the distribution of residences, thereby affecting the residence-based risk of city blocks. It provides empirical and quantitative references for estimating block-level residence-based risk based on RSA development characteristics and for optimizing “residence-based management” plans of epidemic prevention and control. However, residence-based epidemic risk assessment cannot fully reflect the complex infection locations resulting from various population interaction activities, such as those occurring in workplaces, transportation hubs, and leisure areas. Meanwhile, it is also limited in revealing the influence of RSA coverage on block-level infection-location-based risk through its impact on the mixed-use layout of land functions, which is another important characteristic of RSA development. In future studies, we plan to use Location-Based Services (LBS) data, such as signaling data, to assess the epidemic risk of blocks by counting the locations of case intersections and close contacts. Additionally, we will account for the population engaged in different activities within each block. This approach will provide references for estimating block-level infection-location-based risk based on RSA development characteristics and for optimizing prevention and control plans for potential infection sites, thus enabling preemptive judgment and rapid response.

The current planning strategies around RSAs pay more attention to sustainable development of the economy, society, and ecology, rather than to the risk identification and safety enhancement of hazards, especially epidemics. This study contributes to a diverse body of evidence, alongside other retrospective studies, simulation studies, and experimental trials. To sum up, as Rosen (91) pointed out, the COVID-19 pandemic has created a natural experiment of unprecedented proportions, which can offer scientists a chance to try to Test existing hypotheses and Reveal hidden mechanism, Leverage big data, Reveal causality between the possible factors, and Do not stop.

## Data Availability

The raw data supporting the conclusions of this article will be made available by the authors, without undue reservation.
