# Supplementary material for: The effect of urban-rail station area coverage on city blocks’ epidemic transmission: the case of the rail-supportive city of Beijing, China
Source: Front Public Health. 2025 May 30;13:1588004. doi: 10.3389/fpubh.2025.1588004 (PMC12162939; doi:10.3389/fpubh.2025.1588004)
Supplement: Supplementary file 1 [file Data_Sheet_1.docx]

Supplementary Material

# Supplementary Tables

① For testing the total effect of RSA coverage on blocks’ epidemic risk in Section 4.2:

**TABLE S1-1** Case processing summary.

|  | | **Number of Cases** | **Marginal Percentage** |
| --- | --- | --- | --- |
| Etr | 1 | 105 | 78.9% |
|  | 2 | 14 | 10.5% |
|  | 3 | 14 | 10.5% |
| Rcr | high | 48 | 36.1% |
|  | mid | 32 | 24.1% |
|  | low | 53 | 39.8% |
| Valid Cases | | 133 | 100.0% |
| Missing Cases | | 0 |  |
| Total Cases | | 133 |  |

**TABLE S1-2** Model fitting information.

| **Model** | **-2 Log likelihood** | **Chi-square** | **df** | **Sig.** |
| --- | --- | --- | --- | --- |
| Intercept-only Model | 175.714 |  |  |  |
| Final Model | 123.233 | 52.481 | 3 | .000 |

Link function: Logit.

**TABLE S1-3** Parallel lines.

| **Model** | **-2 Log likelihood** | **Chi-square** | **df** | **Sig.** |
| --- | --- | --- | --- | --- |
| parallel lines | 123.233 |  |  |  |
| **General** | 120.138 | 3.095 | 3 | .377 |

Link function: Logit.

② For testing the mediating effect behind the total effect in Section 4.3:

To test the multicollinearity among 8 potential mediating indicators using a variance inflation factor (VIF) analysis：

**TABLE S2** Coefficients.

| **Model** | | **Unstandardized coefficients** | | **Standardized coefficients** | **t** | **Sig.** | **Collinearity statistics** | | |
| --- | --- | --- | --- | --- | --- | --- | --- | --- | --- |
|  |  | **B** | **Std. Error** | **Beta** |  |  | **Tolerance** | **VIF** | |
| 1 | (Constant) | 3.985 | 1.214 |  | 3.284 | .001 |  | |  |
|  | Des | -.162 | .029 | -.493 | -5.646 | .000 | .764 | | 1.309 |
|  | Rps | .103 | .039 | .231 | 2.629 | .010 | .756 | | 1.323 |
|  | Rrp | -1.137 | 1.917 | -.068 | -.593 | .554 | .451 | | 2.219 |
|  | Epp | -6.483 | 6.192 | -.125 | -1.047 | .297 | .410 | | 2.439 |
|  | Jhd | .077 | .155 | .044 | .496 | .620 | .758 | | 1.319 |
|  | Phd | .047 | .248 | .023 | .188 | .851 | .380 | | 2.629 |
|  | Ahd | .009 | .161 | .005 | .056 | .956 | .648 | | 1.544 |
|  | Mrd | -.032 | .050 | -.069 | -.641 | .523 | .508 | | 1.967 |
|  | Efd | -.061 | .101 | -.081 | -.606 | .546 | .326 | | 3.063 |

Dependent Variable: Number of confirmed cases.

To test the effect of Rcr on each mediating indicator using linear regression：

### **TABLE S3-1** Model Summary.

| **Model** | **R** | **R square** | **Adjusted R square** | **Std. error of the estimate** | **Durbin–watson** |
| --- | --- | --- | --- | --- | --- |
| 1 | .365 | .133 | .113 | 4.37489 | 1.866 |

Predictor: (Constant), Rcr, Des.

Dependent Variable: Rps.

**TABLE S3-2** ANOVA.

| **Source** | | **Sum of squares** | **df** | **Mean square** | **F** | **Sig.** |
| --- | --- | --- | --- | --- | --- | --- |
| 1 | \| Regr**ession** \|  \| \| --- \| --- \| | 379.157 | 3 | 126.386 | 6.603 | .000 |
|  | **Residual** | 2469.018 | 129 | 19.140 |  |  |
|  | **Total** | 2848.176 | 132 |  |  |  |

Predictor: (Constant), Rcr, Des.

Dependent Variable: Rps.

### **TABLE S4-1** Model Summary.

| **Model** | **R** | **R square** | **Adjusted R square** | **Std. error of the estimate** | **Durbin–watson** |
| --- | --- | --- | --- | --- | --- |
| 1 | .535 | .286 | .269 | .10499 | 1.458 |

Predictor: (Constant), Rcr, Des.

Dependent Variable: Rrp.

**TABLE S4-2** ANOVA.

| **Source** | | **Sum of squares** | **df** | **Mean square** | **F** | **Sig.** |
| --- | --- | --- | --- | --- | --- | --- |
| 1 | \| **Regression** \| \| --- \| | .565 | 3 | .188 | 17.095 | .000 |
|  | **Residual** | 1.411 | 128 | .011 |  |  |
|  | **Total** | 1.976 | 131 |  |  |  |

Predictor: (Constant), Rcr, Des.

Dependent Variable: Rrp.

**TABLE S5-1** Model Summary.

| **Model** | **R** | **R square** | **Adjusted R square** | **Std. error of the estimate** | **Durbin–watson** |
| --- | --- | --- | --- | --- | --- |
| 1 | .564 | .319 | .303 | .03321 | 1.729 |

Predictor: (Constant), Rcr, Des.

Dependent Variable: Epp.

**TABLE S5-2** ANOVA.

| **Source** | | **Sum of squares** | **df** | **Mean square** | **F** | **Sig.** |
| --- | --- | --- | --- | --- | --- | --- |
| 1 | \| **Regression** \| \| --- \| | .067 | 3 | .022 | 20.104 | .000 |
|  | **Residual** | .142 | 129 | .001 |  |  |
|  | **Total** | .209 | 132 |  |  |  |

Predictor: (Constant), Rcr, Des.

Dependent Variable: Epp.

**TABLE S6-1** Model Summary.

| **Model** | **R** | **R square** | **Adjusted R square** | **Std. error of the estimate** | **Durbin–watson** |
| --- | --- | --- | --- | --- | --- |
| 1 | .452 | .204 | .185 | 1.05505 | 2.240 |

Predictor: (Constant), Rcr, Des.

Dependent Variable: Jhd.

**TABLE S6-2** ANOVA.

| **Source** | | **Sum of squares** | **df** | **Mean square** | **F** | **Sig.** |
| --- | --- | --- | --- | --- | --- | --- |
| 1 | \| **Regression** \| \| --- \| | 36.801 | 3 | 12.267 | 11.020 | .000 |
|  | **Residual** | 143.592 | 129 | 1.113 |  |  |
|  | **Total** | 180.394 | 132 |  |  |  |

Predictor: (Constant), Rcr, Des.

Dependent Variable: Jhd.

### **TABLE S7-1** Model Summary.

| **Model** | **R** | **R square** | **Adjusted R square** | **Std. error of the estimate** | **Durbin–watson** |
| --- | --- | --- | --- | --- | --- |
| 1 | .604 | .365 | .350 | .83069 | 1.823 |

Predictor: (Constant), Rcr, Des.

Dependent Variable: Phd.

**TABLE S7-2** ANOVA.

| **Source** | | **Sum of squares** | **df** | **Mean square** | **F** | **Sig.** |
| --- | --- | --- | --- | --- | --- | --- |
| 1 | \| **Regression** \| \| --- \| | 51.060 | 3 | 17.020 | 24.665 | .000 |
|  | **Residual** | 89.016 | 129 | .690 |  |  |
|  | **Total** | 140.076 | 132 |  |  |  |

Predictor: (Constant), Rcr, Des.

Dependent Variable: Phd.

### **TABLE S8-1** Model Summary.

| **Model** | **R** | **R square** | **Adjusted R square** | **Std. error of the estimate** | **Durbin–watson** |
| --- | --- | --- | --- | --- | --- |
| 1 | .433 | .188 | .169 | 1.11042 | 2.218 |

Predictor: (Constant), Rcr, Des.

Dependent Variable: Ahd.

**TABLE S8-2** ANOVA.

| **Source** | | **Sum of squares** | **df** | **Mean square** | **F** | **Sig.** |
| --- | --- | --- | --- | --- | --- | --- |
| 1 | \| **Regression** \| \| --- \| | 36.765 | 3 | 12.255 | 9.939 | .000 |
|  | **Residual** | 159.062 | 129 | 1.233 |  |  |
|  | **Total** | 195.827 | 132 |  |  |  |

Predictor: (Constant), Rcr, Des.

Dependent Variable: Ahd.

### **TABLE S9-1** Model Summary.

| **Model** | **R** | **R square** | **Adjusted R square** | **Std. error of the estimate** | **Durbin–watson** |
| --- | --- | --- | --- | --- | --- |
| 1 | .621 | .386 | .372 | 3.51822 | 2.362 |

Predictor: (Constant), Rcr, Des.

Dependent Variable: Mrd.

**TABLE S9-2** ANOVA.

| **Source** | | **Sum of squares** | **df** | **Mean square** | **F** | **Sig.** |
| --- | --- | --- | --- | --- | --- | --- |
| 1 | \| **Regression** \| \| --- \| | 1003.984 | 3 | 334.661 | 27.037 | .000 |
|  | **Residual** | 1596.747 | 129 | 12.378 |  |  |
|  | **Total** | 2600.731 | 132 |  |  |  |

Predictor: (Constant), Rcr, Des.

Dependent Variable: Mrd.

### **TABLE S10-1** Model Summary.

| **Model** | **R** | **R square** | **Adjusted R square** | **Std. error of the estimate** | **Durbin–watson** |
| --- | --- | --- | --- | --- | --- |
| 1 | .678 | .459 | .447 | 2.01979 | 1.748 |

Predictor: (Constant), Rcr, Des.

Dependent Variable: Efd.

**TABLE S10-2** ANOVA.

| **Source** | | **Sum of squares** | **df** | **Mean square** | **F** | **Sig.** |
| --- | --- | --- | --- | --- | --- | --- |
| 1 | \| **Regression** \| \| --- \| | 446.825 | 3 | 148.942 | 36.509 | .000 |
|  | **Residual** | 526.261 | 129 | 4.080 |  |  |
|  | **Total** | 973.085 | 132 |  |  |  |

Predictor: (Constant), Rcr, Des.

Dependent Variable: Efd.

To test the effects of Rcr and eight mediating variables on Etr using logistic regression:

**TABLE S11-1** Model fitting information.

| **Model** | **-2 Log likelihood** | **Chi-square** | **df** | **Sig.** |
| --- | --- | --- | --- | --- |
| Intercept-only model | 175.239 |  |  |  |
| Final model | 111.629 | 63.610 | 11 | .000 |

Link function: Logit.

**TABLE S11-2** Parallel lines.

| **Model** | **-2 Log likelihood** | **Chi-square** | **df** | **Sig.** |
| --- | --- | --- | --- | --- |
| parallel lines | 111.629 |  |  |  |
| **General** | 101.366^b^ | 10.263^c^ | 11 | .507 |

**Link function: Logit.**

③ For testing the moderating effect on the mediating effect in Section 4.4:

### **TABLE S12-1** Model Summary.

| **Model** | **R** | **R square** | **Adjusted R square** | **Std. error of the estimate** | **Durbin–watson** |
| --- | --- | --- | --- | --- | --- |
| 1 | .451 | .203 | .165 | 4.24417 | 1.964 |

Predictor: (Constant), Des, Rcr, Rtl, Rcr * Rtl.

Dependent Variable: Rps.

### **TABLE S12-2** ANOVA.

| **Source** | | **Sum of squares** | **df** | **Mean square** | **F** | **Sig.** |
| --- | --- | --- | --- | --- | --- | --- |
| 1 | \| **Regression** \| \| --- \| | 578.538 | 6 | 96.423 | 5.353 | .000^b^ |
|  | **Residual** | 2269.638 | 126 | 18.013 |  |  |
|  | **Total** | 2848.176 | 132 |  |  |  |

Predictor: (Constant), Des, Rcr, Rtl, Rcr * Rtl.

Dependent Variable: Rps.

### **TABLE S13-1** Model Summary.

| **Model** | **R** | **R square** | **Adjusted R square** | **Std. error of the estimate** | **Durbin–watson** |
| --- | --- | --- | --- | --- | --- |
| 1 | .395^a^ | .156 | .116 | 4.36736 | 1.939 |

Predictor: (Constant), Des, Rcr, Rsl, Rcr * Rsl.

Dependent Variable: Rps.

### **TABLE S13-2** ANOVA.

| **Source** | | **Sum of squares** | **df** | **Mean square** | **F** | **Sig.** |
| --- | --- | --- | --- | --- | --- | --- |
| 1 | \| **Regression** \| \| --- \| | 444.870 | 6 | 74.145 | 3.887 | .001^b^ |
|  | **Residual** | 2403.305 | 126 | 19.074 |  |  |
|  | **Total** | 2848.176 | 132 |  |  |  |

Predictor: (Constant), Des, Rcr, Rsl, Rcr * Rsl.

Dependent Variable: Rps.

④ Bootstrap method test on the mediating effect:

**TABLE S14-1** Omnibus test of total effect of X on Y.

| **R2-chng** | **F** | **df1** | **df2** | **p** |
| --- | --- | --- | --- | --- |
| .0565 | 4.6342 | 2.0000 | 128.0000 | .0114 |

**TABLE S14-2** Coding of categorical X variable for analysis.

| **Rcr** | **X1** | **X2** |
| --- | --- | --- |
| 1.000 | .000 | .000 |
| 2.000 | 1.000 | .000 |
| 3.000 | .000 | 1.000 |

**TABLE S14-3** Relative indirect effects of X on Y.

|  |  | **Effect** | **BootSE** | **BootLLCI** | **BootULCI** |
| --- | --- | --- | --- | --- | --- |
| **Rcr→Rps→Ccn** | **X1** | **.2710** | **.2214** | **.0129** | **.8292** |
|  | **X2** | **-.2109** | **.1528** | **-.5828** | **-.0155** |
| Rcr→Rrp→Ccn | X1 | -.0605 | .1369 | -.3845 | .1786 |
|  | X2 | -.1294 | .2765 | -.7580 | .3568 |
| Rcr→Epp→BCcn | X1 | -.1110 | .1067 | -.3486 | .0698 |
|  | X2 | -.2115 | .1872 | -.6044 | .1355 |
| Rcr→Jhd→Ccn | X1 | .0618 | .0831 | -.0658 | .2606 |
|  | X2 | .1425 | .1797 | -.1445 | .5455 |
| Rcr→Phd→Ccn | X1 | .0258 | .1994 | -.4388 | .3443 |
|  | X2 | .0460 | .3530 | -.7440 | .6078 |
| Rcr→Ahd→Ccn | X1 | .0042 | .0680 | -.1499 | .1253 |
|  | X2 | .0077 | .1129 | -.2382 | .2047 |
| Rcr→Mrd→Ccn | X1 | -.0627 | .1522 | -.4356 | .1960 |
|  | X2 | -.0827 | .1931 | -.5455 | .2633 |
| Rcr→Efd→Ccn | X1 | -.0636 | .1584 | -.3224 | .3132 |
|  | X2 | -.1247 | .3067 | -.6304 | .5899 |

Note: If the Bootstrap confidence interval does not include zero, the mediating effect is considered significant; conversely, if it includes zero, the mediating effect is not significant.

**TABLE S14-4** Omnibus test of direct effect of X on Y.

| **R2-chng** | **F** | **df1** | **df2** | **p** |
| --- | --- | --- | --- | --- |
| .0076 | .6436 | 2.0000 | 120.0000 | .5272 |
